# Polypharmacy and drug-related problems among people living with HIV/AIDS: a single-center experience

**DOI:** 10.3906/sag-1807-295

**Published:** 2019-02-11

**Authors:** Emre KARA, Ahmet Çağkan İNKAYA, Duygu AYDIN HAKLI, Kutay DEMİRKAN, Serhat ÜNAL

**Affiliations:** 1 Department of Clinical Pharmacy, Faculty of Pharmacy, Hacettepe University, Ankara Turkey; 2 Department of Infectious Diseases and Clinical Microbiology, Hacettepe University Hospitals, Ankara Turkey; 3 Department of Biostatistics, Faculty of Medicine, Hacettepe University, Ankara Turkey

**Keywords:** Human immunodeficiency virus, polypharmacy, drug-related problems

## Abstract

**Background/aim:**

The HIV-infected population is aging, and the concomitant comorbidities increase the likelihood of polypharmacy. There is a scarcity of data for determining drug-related problems in people living with HIV/AIDS (PLWHA).

**Materials and methods:**

This cross-sectional study was carried out between 1 September 2015 and 1 July 2016. All patients underwent a face-to-face interview with a clinical pharmacist. PCNE Classification V 7.0 was used classify incident drug-related problems (DRPs).

**Results:**

The mean age of the patients was 40.4 ± 13.06 years. The rate of polypharmacy was 66.1% in patients with comorbidities and 12.3% in those without comorbidities (P < 0.001). DRPs were more prominent in older patients (46 vs. 37 years, P < 0.001), those with longer durations of antiretroviral therapy (ART) (45 vs. 27 months, P = 0.014), and those with lower education levels (P = 0.013). Receiving >3 ART drugs was associated with more DRPs in the logistic regression model (odds ratio: 8.299, 95% confidence interval: 1.924–35.803). Fifty-eight interventions were performed in 45 (24.9%) patients. Clinical pharmacist interventions were performed in 18.9% of patients without polypharmacy and in 38.9% of patients with polypharmacy (P < 0.001).

**Conclusion:**

DRPs and polypharmacy are common among elderly PLWHA. More interventions are warranted to boost the quality of life in aging PLWHA.

## 1. Introduction

According to the World Health Organization, 1 out of 200 people were living with human immunodeficiency virus/acquired immune deficiency syndrome (HIV/AIDS) in 2015, and four new infections occurred each minute (http://www.unaids.org/sites/default/files/media_asset/global-AIDS-update-2016_en.pdf). The first HIV/AIDS case in Turkey was reported in 1985 and more than 12,000 people have been diagnosed since then (1). The Turkish HIV epidemic has recently expanded at an accelerated rate and the number of new HIV diagnoses has increased by 450% after 2010 (https://www.saglik.gov.tr).

Life expectancy in HIV-infected populations is approaching that of uninfected populations, and the difference is getting smaller every year (2). People living with HIV/AIDS (PLWHA) are destined to receive multiple medications to suppress viral replication, as well as to manage/treat concomitant diseases. As lifelong treatment is required, it is fundamental to provide safe and effective pharmacotherapy for all patients until a cure is possible (3). 

In addition to aging with HIV, HIV prevalence among older individuals is also increasing. Secondary to aging with HIV, the number of concomitant chronic diseases and associated medications increases, which eventually leads to polypharmacy (4).

Polypharmacy is defined as the use of five or more u2905 by a u2908. Polypharmacy is a growing concern among PLWHA because of adverse drug reactions (ADRs), drug–drug interactions, reduced adherence, and reduced treatment tolerance (5). The risk of polypharmacy and thus the likelihood of drug-related problems (DRPs) increases with the number of concomitant diseases in PLWHA (4). 

According to the Pharmaceutical Care Network Europe (PCNE), a DRP is “an event or circumstance involving drug therapy that actually or potentially interferes with desired health outcomes” (https://www.pcne.org/working-groups/2/drug-related-problems). Classification systems other than that of the PCNE have been created by different research groups based on the choice of drug, drug dosages, ADRs, drug interactions, lack of monitoring of drug effects/toxicity, and adherence problems (6). The PCNE classification has been implemented in the management of heart failure and diabetes mellitus to determine drug-related problems. Studies have confirmed the frequent existence of DRPs in patients with chronic conditions and have highlighted the unique role of the PCNE in the evaluation of DRPs. The PCNE allows for the evaluation of clinical pharmacist interventions and associated outcomes (7). The aim of this study was to determine DRPs and to evaluate clinical pharmacist interventions among PLWHA followed at a university hospital.

## 2. Materials and methods

This cross-sectional study was carried out between 1 September 2015 and 1 July 2016 at the Infectious Diseases Outpatient Clinic of the Hacettepe University Adult Hospital. All PLWHA attending routine outpatient follow-up visits were informed about the study and were invited to participate. To eliminate selection bias, all PLWHA attending routine outpatient follow-up visits were informed and invited. Inclusion criteria were age 18 years or older, under ART for at least 3 months, and not involved in any clinical trial during the study period. All PLWHA who provided informed consent were included. Demographic data of the patients, laboratory findings, medications used, and other nondrug product data were collected. Each patient underwent a complete medical follow-up (supervised by board-certified physicians) and routine blood tests. A clinical pharmacist then interviewed the patients who had provided informed consent. Clinical pharmacists provided detailed information on the usage of ARTs, drug–drug interactions, drug–food interactions, and key aspects of ART. Medications prescribed by physicians as well as those used without medical advice were recorded. All medications were defined according to the active chemical moiety (e.g., Truvada = tenofovir disoproxil fumarate plus emtricitabine). Data on ART regimen, ART adherence, the virological and immunological success of the treatment, concurrent diseases, and concomitant medications were recorded for all participating PLWHA. Nucleus software (utilized for the management of patients by Hacettepe Hospitals) and patient files were used to collect data. Potential drug–drug interactions were identified by using the online Micromedex Solutions software and the www.hiv-druginteractions.org database (8). It is generally accepted that consumption of 5 different medications is the threshold associated with negative health outcomes, and co-medication with 5 or more drugs has been classified as polypharmacy (9). The clinical pharmacist’s final evaluation was made using current guidelines and online data sources (including but not limited to the European AIDS Clinical Society Guidelines, United States Department of Health and Human Services Guidelines, and the Turkish Ministry of Health Guidelines). After the pharmacist interview, an official recommendation was offered to the attending physician and the participant, which encompassed treatment, drug–drug interactions, drug–disease interactions, side effects associated with antiretroviral drugs, drug abuse, and prescribing errors. PCNE Classification V 7.0 was used to classify the findings pertaining to problems, causes, recommendations, and recommendations related to drugs (http://europharm.pbworks.com/w/file/113186797/PCNE%20classification%20V7-0.pdf).

The study protocol was evaluated and approved by the Hacettepe University Non-Interventional Ethics Committee (decision number: GO-15/558-14).

Quantitative data are summarized as mean (±standard deviation) and median (minimum–maximum); frequency and percentages are presented for qualitative data. The normality of each variable was tested using the Kolmogorov–Smirnov test. The independent t-test was used to compare two groups with normal distribution, and the Mann–Whitney U test was used for variables with nonnormal distribution. Logistic regression was used to determine the association between DRPs and age, duration of treatment, CD4+ cell count, education level, number of additional diseases, and number of medications. The data are summarized by box plot line, scatter, and line graphs. IBM SPSS 23.0 was used to evaluate the data. P < 0.05 was considered statistically significant for all tests.

## 3. Results 

From 1998 until the end of the study period, 450 patients were registered in the Hacettepe cohort. However, only 320 of the 450 patients were under regular follow-up. Among the patients who attended their routine follow-ups, 190 were invited to participate in the study. Four refused to participate and 186 were enrolled. Among those enrolled, 5 patients were excluded (2 patients withdrew informed consent, 2 were not under ART, and 1 patient had cognitive issues and could not answer the questions) and the remaining 181 patients were included in final analysis. The mean age of the patients was 40.4 ± 13.1 years (range: 18–70 years). The demographic and infection-related characteristics of the cohort are summarized in Tables 1 and 2. A majority (70%) of the study participants were diagnosed after 2011.

**Table:** Patient demographics.

Age, mean ± SD	40.4 ± 13.06
<50	134 (74%)
≥50	47 (26%)
Sex, n (%)	
Male	144 (79.6%)
Female	37 (20.4%)
Marital status, n (%)	
Single	92 (50.8%)
Married	89 (49.2%)
Body mass index (BMI)	
<18.5 kg/m2	4 (2.2%)
18.5–24.9 kg/m2	92 (50.8%)
25–29.9 kg/m2	65 (35.9%)
>30 kg/m2	20 (11.1%)
Smoking, n (%)	
Smoker	96 (53%)
Nonsmoker	85 (47%)
Alcohol, n (%)	
User	62 (34.3%)
Nonuser	119 (65.7%)
Education, n (%)	
No education	4 (2.2%)
Primary school	31 (17.1%)
Secondary school	19 (10.5%)
High school	41 (22.7%)
University	63 (34.8%)
Postgraduate	23 (12.7%)

SD: Standard deviation.

**Table 2:** HIV infection and antiretroviral treatment-related data.

CD4+ T lymphocyte count (cell/mm3), median (min–max)	586 (4–1740)
Nadir CD4+ T lymphocyte count (cell/mm3), median (min–max)	309 (4–928)
Viral loads, <40/mL, n (%)	145 (80.1%)
40–200/mL, n (%)	13 (7.2%)
>200/mL, n (%)	23 (12.7%)
Viral load (copies/mL), median (min–max)	299 (42–138,250)
Antiretroviral drug classes, n (%)	
NRTI	179 (98.9%)
Integrase inhibitors	92 (50.8%)
Protease inhibitors	61 (33.7%)
NNRTI	39 (21.5%)
Entry inhibitors	2 (1.1%)

NRTI: Nucleoside reverse transcriptase inhibitors, NNRTI: nonnucleoside reverse transcriptase inhibitors.

### 3.1. Comorbidities

One hundred and twenty-two participants (67.4%) did not have any comorbidities, whereas 59 (32.6%) presented with at least one comorbidity. Comorbidities included hypertension (12.7%), dyslipidemia (9.4%), major depression (7.7%), diabetes (7.2%), coronary artery disease (3.9%), and other diseases (such as osteoporosis, hypothyroidism, asthma, and epilepsy) (12.4%). Thirty-six (19.9%), 11 (6.1%), and 12 (6.6%) of the participants had 1, 2, and 3 comorbidities, respectively. 

### 3.2. Medications used by patients

A total of 790 medications used by participants were recorded; 644 (81.5%) of these drugs were ARTs, and the remaining 146 (18.5%) were medications other than ARTs. ART regimens included the following: nucleoside/nucleotide reverse transcriptase inhibitor (NRTI) + integrase strand transfer inhibitor (INSTI) (44.3%), NRTI + protease inhibitor (PI) (28.7%), NRTI + nonnucleoside reverse transcriptase inhibitor (NNRTI) (19.9%), and other regimens (7.1%). All patients received combined ART consisting of a combination of at least 3 active antiretroviral drugs. The most common ART regime used was an integrase inhibitor-based regime (44.3%). Eight patients (4.4%) received 5 active ART-containing regimes. Sixty-eight percent of the patients did not receive any co-medication other than the ART drugs, while 8.9% received at least 4 different medications other than the ART drugs. Eleven percent of the patients received at least 6 medications in total (Table 3). 

**Table 3:** Number of drugs used by patients.

Number of drugs	Antiretrovirals n (%)	Co-medication n (%)	Antiretroviral plus co-medication n (%)
0	-	123 (68.0%)	-
1	-	23 (12.7%)	-
2	-	12 (6.5%)	-
3	88 (48.6%)	6 (3.3%)	53 (29.3%)
4	85 (47.0%)	9 (5.0%)	74 (40.8%)
5	8 (4.4%)	4 (2.2%)	27 (14.9%)
6	-	3 (1.7%)	7 (3.9%)
7	-	1 (0.6%)	11 (6.1%)
8	-	-	2 (1.1%)
9	-	-	5 (2.8%)
10	-	-	2 (1.1%)

### 3.3. Polypharmacy, drug-related problems, and clinical pharmacist intervention

Overall, polypharmacy was detected in 29.9% of the patients. The rate of polypharmacy was 12.3% in those without comorbidities, while it increased to 66.1% in those with comorbidities (P < 0.001). In our study, polypharmacy was associated with age (mean: 37.17 ± 11.84 vs. 48.00 ± 12.71 years, P < 0.001), nadir CD4 [median: 323 (10–928) vs. 255 (4–918) cell/mm3, P = 0.03], duration of ART [median: 27 (3–320) vs. 46 (6–236) months, P = 0.009], intensive treatment and integrase inhibitor-based treatment (P < 0.001), and diseases of the cardiovascular system (CVS) (P < 0.001) and the central nervous system (CNS) (P < 0.001). Fifty-eight DRPs were found in 45 patients. DRPs were more prominent with advanced age (46 vs. 37 years, P < 0.001), longer durations of ART (45 vs. 27 months, P = 0.014), and lower education level (P = 0.013). Patients receiving intensive ART (>3 ART drugs) had more DRPs in the logistic regression model (odds ratio (OR): 8.299, 95% confidence interval (CI): 1.924–35.803) (Table 4). Patients receiving intensive ART are at risk of experiencing about 8 times more DRPs than are other patients. Fifty-eight interventions were performed in 45 patients. Twenty-nine (50%) of the interventions were geared towards the physician alone, 25 (43%) towards the patient alone, and 4 were geared towards both the physician and the patient. Twenty-nine (50%) of the interventions involved comorbidities and co-medications, 19 (32.8%) involved antiretroviral drugs, 7 involved antiretroviral drugs and co-medications (6 orange ‘potential interactions’ and 1 red ‘do not co-administer’ interaction according to the www.hiv-druginteractions.org database), and 3 involved drugs used for prophylaxis. Clinical pharmacist interventions were performed in 22.1% of the patients without comorbidities and in 30.5% of those with one or more comorbidities (P = 0.227). Clinical pharmacist interventions were performed in 18.9% of the patients without polypharmacy and in 38.9% of patients with polypharmacy (P < 0.001). Twenty-nine (50%) interventions involved comorbidities or co-medications and 19 (32.8%) of these involved ART or antiretroviral drugs. Problems, causes, interventions, implementations, and outcomes were classified as per PCNE classifications (Table 5). 

**Table 4:** Drug-related problems in people living with HIV/AIDS under treatment.

		DRP (+)	DRP (-)	P-value
Age, median (min–max) (years)		46 (23–71)	37 (18–74)	<0.001
Nadir CD4, mean (min–max) (cells/mm3)		278 (10–928)	313.5 (4–856)	0.136
Current CD4, mean (min–max) (cells/mm3)		595 (92–1740)	585 (4–1274)	0.611
Duration of therapy, median (min–max) (months)		45 (6–229)	27 (3–320)	0.014
Sex, n (%)	Female	8 (17.8%)	29 (21.3%)	0.635
	Male	37 (82.2%)	107 (78.7%)	
Education level, n (%)	Primary school	15 (33.3%)	20 (14.7%)	0.013
	Secondary school	4 (8.9%)	15 (11%)	
	High school	11 (24.4%)	29 (21.3%)	
	University	14 (31.1%)	51 (37.5%)	
	Postgraduate	1 (2.2%)	21 (15.4%)	
Smoking, n (%)	Smoker	17 (37.8%)	56 (41.2%)	0.738
	Nonsmoker	28 (62.2)	80 (58.8%)	
ART regimen, n (%)	PI based	5 (11.1%)	47 (34.6%)	<0.001
	NNRTI based	14 (31.1%)	22 (16.2%)	
	Integrase inhibitor-based	19 (42.2%)	61 (44.9%)	
	Intensive	7 (15.6%)	6 (4.4%)	
Comorbidities, n (%)	CVS diseases	14 (58.3%)	22 (47.8%)	0.036
	CNS diseases	6 (25%)	10 (21.7%)	0.240
	Endocrine diseases	3 (12.5%)	5 (10.9%)	0.418
	Other diseases	1 (4.2%)	9 (19.6%)	0.221

DRP: Drug-related problem, ART: antiretroviral therapy, NNRTI: nonnucleoside reverse transcriptase inhibitors, CVS: cardiovascular system, CNS: central nervous system, PI: protease inhibitors.

**Table 5:** Classification of interventions according to Pharmaceutical Care Network Europe (PCNE) classification.

PCNE Classifications	Frequency (%)	
Problem		
P1.1. No effect of drug treatment/therapy failure	1	1.7%
P1.2. Effect of drug treatment not optimal	26	44.8%
P1.3. Unnecessary drug-treatment	1	1.7%
P1.4. Untreated indication	19	32.8%
P2.1. Adverse drug event occurrence	9	15.5%
P3.1. Patient dissatisfied with therapy despite optimal clinical and economic treatment outcomes	2	3.4%
Cause		
C1.1. Inappropriate drug according to guidelines/formulary	1	1.7%
C1.2. Inappropriate drug (within guidelines but otherwise contraindicated)	10	17.2%
C1.3. No indication for drug	1	1.7%
C1.4. Inappropriate combination of drugs, or drugs and food	9	15.5%
C1.6. Indication for drug-treatment not noticed	11	19.0%
C1.9. New indication for drug treatment presented	1	1.7%
C3.1. Drug dose too low	1	1.7%
C3.2. Drug dose too high	5	8.6%
C6.1. Inappropriate timing of administration and/or dosing intervals	6	10.3%
C6.3. Drug overadministered	1	1.7%
C6.4. Drug not administered at all	7	12.1%
C7.1. Patient forgets to use/take drug	2	3.4%
C7.8. Patient unable to use drug/form as directed	3	5.2%
Intervention		
I1.1. Prescriber informed only	5	8.6%
I2.1. Patient (drug) counseling	5	8.6%
I3.1. Drug changed	11	19.0%
I3.2. Dosage changed	6	10.3%
I3.4. Instructions for use changed	11	19.0%
I3.5. Drug stopped	3	5.2%
I3.6. New drug started	17	29.3%
Implementation		
A1.1. Intervention accepted and fully implemented	51	87.9%
A1.2. Intervention accepted, partially implemented	1	1.7%
A1.4. Intervention accepted, implementation unknown	3	5.2%
A2.1. Intervention not accepted: not feasible	3	5.2%
Outcome of intervention		
O0.0. Problem status unknown	5	8.6%
O1.0. Problem totally solved	50	86.2%
O3.4. No need or possibility to solve problem	3	5.2%

PCNE: Pharmaceutical Care Network Europe.

## 4. Discussion

Our findings suggest that DRPs and polypharmacy are common in the outpatient setting. A number of studies previously evaluated DRPs in HIV-infected patients during hospital admission. It was reported that inappropriate amounts and frequencies of dosing and dose omission were the most common mistakes occurring during hospital admissions (10,11). 

Polypharmacy causes adverse outcomes such as drug interactions and reduction in drug compliance (4). Polypharmacy can lead to nonadherence, adverse drug reactions, drug–drug interactions, geriatric syndromes (falls, fractures, and dementia), and increased prescription errors, morbidity, and mortality (12–14). A study by Krentz et al. showed that daily pill burden and as well as polypharmacy decreased as a result of advances in ART between 1998 and 2010 (15). Our study group consisted of relatively newly diagnosed PLWHA. Despite the recent advances in ART, polypharmacy was found to be present in this PLWHA cohort. Holtzman et al. found that ³5 medications and ³5 non-antiretroviral medications were used in 61% and 39% of the patients, respectively (16). The usage of co-medication was found to be higher in the elderly (³50 years) in a study that analyzed patients receiving combined ARTs. Also, in patients ³50 years old, usage of one or more and five or more co-medications was detected in 82% and 58% of the patients, respectively (13). Tseng et al. found that older patients (≥50 years) used more drugs than did younger patients (17). In our study, polypharmacy was associated with age, nadir CD4+, duration of ART, intensive treatment and integrase inhibitor-based treatment, and CVS and CNS diseases. Also, overall polypharmacy was found in 29.9% of our study group. The rate of polypharmacy was 12.3% in those without comorbidities, while it increased to 66.1% in those with comorbidities (P < 0.001). In order to overcome the issues associated with polypharmacy, mono and dual therapies should be evaluated head-to-head in PLWHA with comorbidities. 

In a study by Molino et al., drug-related problems decreased from 5.2 to 4.2 per patient (P = 0.043) (18). Silveira et al. evaluated 319 patients for 1 year and found 94 drug-related problems in 161 intervention group patients; 43% of the problems were resolved. The majority of the identified problems (53%) were related to either using nonprescription medications or not using prescribed medications (19). Foisy et al. found 149 drug-related problems and resolved more than 95% of the problems by following 57 patients for 14 months. Among the problems identified, adverse reactions and drug interactions were the most common (20).

In our study, DRPs were more prominent with advanced age, longer duration of ART, and lower education level. Patients receiving intensive ART had more DRPs in the logistic regression model. One of the earliest studies investigating the participation of the pharmacist in the ART process was conducted by Walji et al. in 1989. In this study, 285 AIDS patients who received zidovudine treatment were evaluated and 97% of the interventions were accepted (21). In 1998, Bozek et al. found 4.6 clinical care activities per patient in HIV-infected patients and 1.9 in noninfected patients (P < 0.005). Common drug-related problems in this study were drug without indications (15%), overdose (13%), and adverse drug reactions (13%) (22). Eginger et al. detected 210 problems in highly active antiretroviral therapy (HAART) and opportunistic infection prophylaxis, 172 of which were HAART-related, while the rest were associated with prophylaxis. At least one drug error was detected in 54.7% of the patients. The acceptance rate of the pharmacist’s interventions for DRPs by physicians was 90%. Problems with antiretroviral drugs were missing dose, incorrect dose, incomplete regimen, incorrect regimen, no dose adjustment for renal function, drug–drug interaction, duplication, and no dose adjustment for liver function (10). In 2007, Carcelero et al. assessed 247 prescriptions of 189 patients and 60 antiretroviral-related problems were detected in 41 patients. A majority (91.7%) of the pharmacist interventions for the problems identified during the study were accepted by the physicians (23). Billedo et al. showed that the clinical pharmacist’s participation in the Antiretroviral Stewardship Program contributed to a reduction of drug-related errors and improvement of drug safety in patients (24). Evaluation of patients’ treatments and recommendations to physicians and patients during ART by pharmacists have been reported by numerous studies in literature (10,22,25). 

In our study, half of the interventions were geared towards the physicians, 43% towards the patients, and 7% towards both the physicians and the patients. More clinical pharmacist interventions were performed in patients with one or more comorbidities and in patients with polypharmacy. In this study 93.2% of the interventions were accepted by the physicians. The number of patients and the average age of patients is projected to increase gradually in this population. As a result, the number of drugs used by patients will increase; in addition, interventions and contributions of the pharmacist to the patient treatment are also likely to increase. 

Our study has some limitations. First, this is a single-center study carried out at a tertiary referral center in central Turkey. Due to the nature of the referral center (consultations offered for follow-up), many patients appear at a single visit and return to their respective medical centers for further follow-up. We only recruited patients who were followed exclusively in our unit. The second limitation is the overrepresentation of newly diagnosed patients in the cohort. The Turkish HIV epidemic has recently expanded into an accelerating phase and more than half of the patients diagnosed so far have been diagnosed in the last 4 years. Another limitation of this study was its cross-sectional design. 

On the other hand, this is the first study evaluating PCNE classification in the follow-up of PLWHA. Furthermore, to our knowledge, this is the first study in a low endemic country describing polypharmacy and DRPs among PLWHA. 

Drug-related problems and polypharmacy are common among Turkish PLWHA, and these conditions increase the likelihood of drug interactions, side effects, and dose adjustment. Though polypharmacy may also be present in younger PLWHA, special attention should be paid to the elderly and heavily treated PLWHA. As the infected population gets older, more interventions are warranted to support the quality of life in PLWHA. Clinical pharmacists could potentially be included in multidisciplinary patient care teams, where they may contribute to reducing the workload of physicians and to improving treatment outcomes by sharing information and interventions. In addition, they may contribute to the outcome of treatment by counseling patients about the proper use of pharmaceutical drugs.

## Acknowledgments

The authors are honored to express their sincere gratitude to the patients who voluntarily contributed to this study.
